# Citalopram in the treatment of elderly chronic heart failure combined with depression: A systematic review and meta-analysis

**DOI:** 10.3389/fcvm.2023.1107672

**Published:** 2023-02-01

**Authors:** Longmei Yan, Yuzhen Ai, Yaxuan Xing, Biqing Wang, Anran Gao, Qiwu Xu, Hongzheng Li, Keji Chen, Jingchun Zhang

**Affiliations:** ^1^Xiyuan Hospital, China Academy of Chinese Medical Sciences, Beijing, China; ^2^National Clinical Research Center for Chinese Medicine Cardiology, Xiyuan Hospital, China Academy of Chinese Medical Sciences, Beijing, China; ^3^Graduate School, Beijing University of Chinese Medicine, Beijing, China; ^4^The Second Affiliated Hospital of Guizhou University of Traditional Chinese Medicine, Guizhou, China

**Keywords:** citalopram, elderly chronic heart failure, depression, systematic review, meta-analysis

## Abstract

**Background:**

Depression is an independent factor to predict the hospitalization and mortality in the chronic HF patients. Citalopram is known as an effective drug for depression treatment. Currently, there is no specific recommendation in the HF guidelines for the treatment of psychological comorbidity. In recent years, many studies have shown that the citalopram may be safe in treating of chronic HF with depression.

**Objective:**

To evaluate the efficacy and safety of the citalopram in the treatment of elderly chronic HF combined with depression.

**Methods:**

PubMed, EMBASE, Cochrane, Web of Science, CNKI, VIP, CBM, and Wanfang were searched from their inception to May 2022. In the treatment of elderly chronic HF combined with depression, randomized controlled studies of the citalopram were included. Independent screening and extraction of data information were conducted by two researchers, and the quality was assessed by the Cochrane bias risk assessment tool. Review manager 5.4.1 was employed for statistical analysis.

**Results:**

The results of meta-analysis prove that the citalopram treatment for depressed patients with chronic HF has a benefit for HAMD-24 (MD: −8.51, 95% CI: −10.15 to −6.88) and LVEF (MD: 2.42, 95% CI: 0.51 to 4.33). Moreover, the score of GDS decreases, and NT-proBNP (MD: −537.78, 95% CI: −718.03 to −357.54) is improved. However, the comparison with the control group indicates that there is no good effect on HAMD-17 (MD: −5.14, 95% CI: −11.60 to 1.32), MADRS (MD: −1.57, 95% CI: −3.47 to 0.32) and LVEDD (MD: −1.45, 95% CI: −3.65 to −0.76). No obvious adverse drug reactions were observed.

**Conclusion:**

Citalopram treatment for depressed patients with chronic HF has a positive effect on LVEF and NT-proBNP. It can alleviate HAMD-24 and GDS, but the relative benefits for LVEDD, HAMD-17 and MADRS still need to be verified.

**Systematic Review Registration**: PROSPERO [CRD42021289917].

## 1. Introduction

Heart failure (HF) is the end stage of various cardiovascular diseases. Due to the disease and psychological pressure, experience depression may prolonged duration of illness. The prevalence of depression is significantly higher in HF patients than that in the general population (1–3). The incidence of HF combined with depression ranged from 31.0 to 77.5% (4). As the severity of HF increases, the incidence of depression increases (3). It leads to the frequent hospitalization, high medical expenses, heavy burden to family and social medical treatment, which seriously reduces the life quality of patients (5).

Cardiovascular disease combined with psychological problems has attracted the increasing attention. In 2014, the American Heart Association issued a scientific statement that depression was one of the risk factors for heart disease (6). Depression can increase the morbidity and mortality of heart disease (7), while the mechanism is not clear. Depression is extremely common prevalent in HF patients (8), which is an independent predictor of hospitalization and mortality in HF patients (9). The European Society of Cardiology (ESC) and American College of Cardiology/American Heart Association (ACC/AHA) HF guidelines recommend screening and treating depression in HF patients (10, 11). Due to the overlap between cardiac and psychological symptoms, multiple challenges are encountered in the recognition and management of depression in patients with HF. Currently, HF guidelines are deficient in the management of depression (12). And the effectiveness of antidepressant therapy on the outcome for patients with chronic HF and depression is controversial.

Citalopram is a selective serotonin reuptake inhibitors (SSRIs), composed of two enantiomers, R-citalopram and S-citalopram, which exerts an antidepressant effect by inhibiting the neurological reuptake of 5-HT (13). As for Escitalopram (the S-(+)-enantiomer of citalopram), the inhibitory function on 5-HTT is approximately twice more than that of the citalopram (14). It has a good curative effect and patient acceptability (15). SSRIs can lead to QTc prolongation, the citalopram and escitalopram have also been confirmed as being responsible for QTc prolongation and thus the danger of possible arrhythmias (16, 17). It should be noted that SSRIs do cause the QTc prolongation, to a much lower degree than the older tricyclic antidepressants (TCAs) (17). As such, SSRIs appear to be a safe treatment option for depression in chronic HF patients (18). Depression can aggravate the patient progress, while the HF can further aggravate the depression. Regarding the patients with chronic HF and depression, we can learn from complex interplay of cardiac physiology and social psychology. The researches of depression and HF are very complex. However, there are a number of pathophysiological interaction mechanisms which are important in exploring available ways to alleviate the depression and improve cardiac function.

To date, there is no evidence that antidepressant treatment can improve the symptoms in patients with chronic HF, and the HF guidelines do not contain specific recommendations for treating psychological comorbidity (12). This study conducted a meta-analysis of data from domestic and international clinical trials to evaluate whether the citalopram is a safe and effective treatment for chronic HF with depression, which may provide a reference for clinical practice and research.

## 2. Materials and methods

### 2.1. Protocol and registration

This systematic review is abided by the Preferred Reporting Items for Systematic Reviews and Meta-Analysis (PRISMA) statement (19). The study was registered at the PROSPERO (ID CRD 42021289917) in December 2021.

### 2.2. Search strategies

An article search was carried out in the following eight databases from its establishment to May 2022, including PubMed, Web of Science, Cochrane Library, Embase, CNKI, VIP, CBM, and Wanfang. The language was restricted to Chinese and English. In these databases, “citalopram,” “heart failure” and “depression” were used as the subject words. The combination of MeSH keywords and free words were used for the search. We also searched the conference abstracts, dissertations and other grey papers. Manual retrieval of all references was summarized in review. Specific retrieval strategy was presented in Supplementary Table 1, and the last retrieval was conducted in 2022.

### 2.3. Inclusion criteria

The following studies were included in the meta-study: (1) Type of study: randomized controlled trials (RCTs); (2) Study object: included participants were adults aged 60 or older; (3) Content standard: met the diagnostic criteria for HF and depression, Diagnostic criteria for HF: met Chronic Heart Failure (20) or met New York Heart Association class (NYHA). Diagnostic criteria for depression: no clear standardized diagnostic criteria for depression, met diagnostic criteria for depression, such as (CCMD-3, DSM-IV, and ICD-10), or assessed with any validated depression scale (HAMD-17, HAMD-24, GDS, and MARDS); (4) Intervention measure: citalopram was used as an intervention drug (dosage form and manufacturer were not limited); and (5) Outcome indicators: Depression score (HAMD-17, HAMD-24, GDS, and MARDS) and Left ventricular ejection fractions (LVEF) as the primary outcome indicators; Left ventricular end diastolic diameter (LVEDD), N-terminal prohormone of brain natriuretic peptide (NT-proBNP) as the primary outcome indicators; adverse reactions such as nausea, vomiting, dizziness, fatigue, and insomnia were safety outcome indicators.

### 2.4. Exclusion criteria

Studies meeting the following criteria were excluded: (1) duplicated publications; (2) animal studies, research protocols and review articles; (3) the use of any other herbal medicines during the research; and (4) the use of antidepressants other than the citalopram.

### 2.5. Data extraction

Two researchers (LY and XY) independently screened and extracted the data according to the inclusion and exclusion criteria. Then their results were cross-checked. Any disagreements were resolved by a third party (BW).

### 2.6. Quality assessment

The risk bias assessment tool recommended by the Cochrane Collaboration was used to evaluate the quality of included literature. Several bias were successively assessed, such as Random sequence generation (selection bias), allocation concealment (selection bias), blinding of participants and personnel (performance bias), blinding of outcome assessment (detection bias), incomplete outcome date (attrition bias), selective reporting (reporting bias) and other. Risk bias was assessed for each project through three levels: “low risk, unclear and high risk.”

### 2.7. Statistical analysis

Review manager 5.4.1 was used for data meta-analysis. *Q* test and *I*^2^ value were used to evaluate the heterogeneity among the included studies. If *p* > 0.1 and *I*^2^ < 50%, the heterogeneity across studies were considered relatively small, and a fixed effect model was adopted; otherwise, a random effect model was used. For higher heterogeneity, subgroup analyses were conducted to explore the sources of heterogeneity. A sensitivity analysis was performed to check the result stability. More than 10 trials were included, publication bias would be evaluated by funnel plot.

## 3. Results

### 3.1. Included studies characteristics

**Figure 1:**
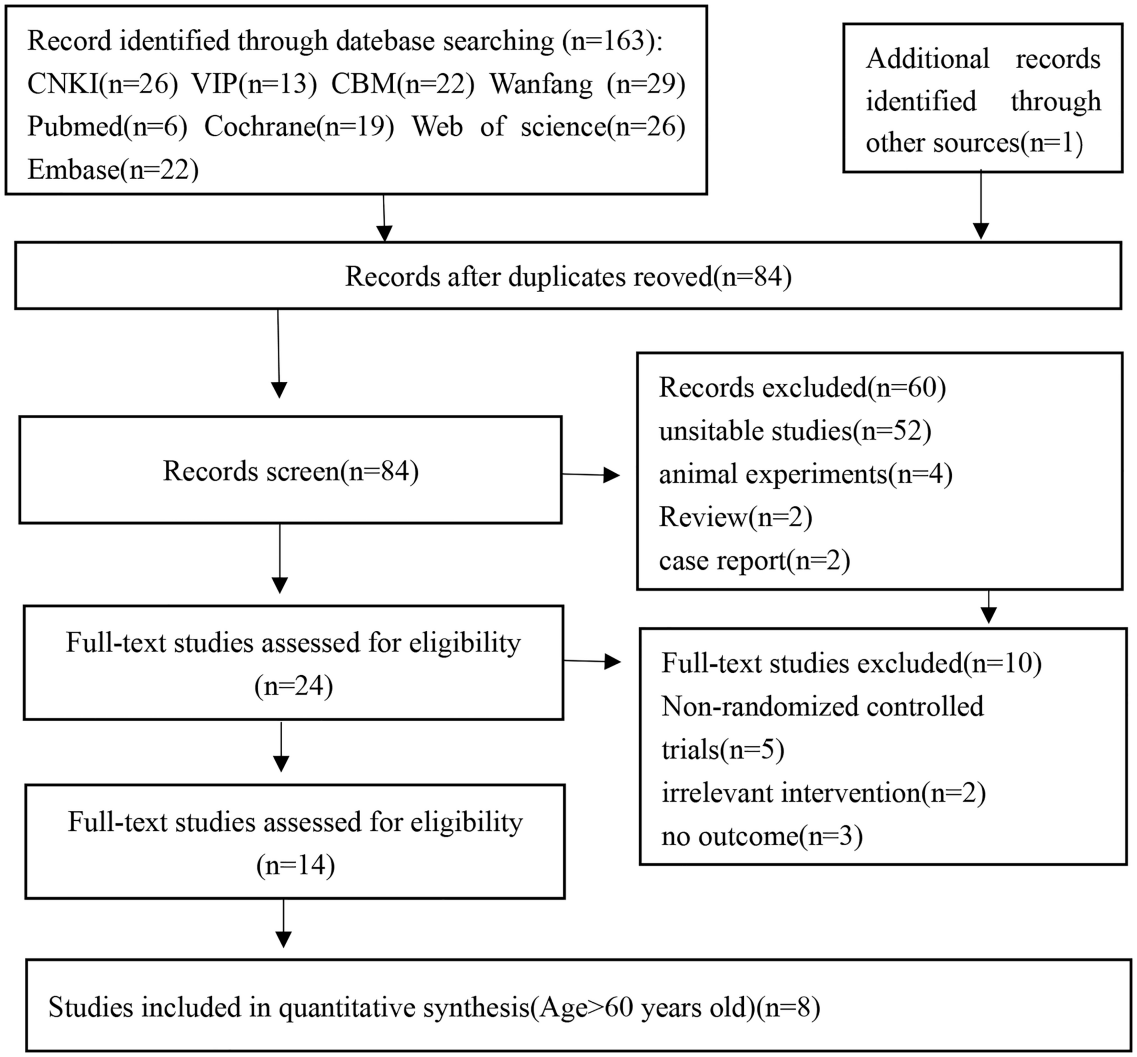
Flow diagram of the literature selection.

A total of 164 studies were retrieved through the search strategy. After excluding 84 duplications, the remaining studies were screened based on their titles and abstracts, and 60 irrelevant studies were removed. Ten studies were excluded by reading the full text. Six articles were excluded due to age less than 60. Finally, eight randomized controlled studies were included in the meta-analysis (Figure 1).

A total of eight studies met the inclusion criteria, involving 1,141 patients (573 experimental and 568 control). The age of all the participants was over 60 years. These studies were carried out from 2011 to 2020. The basic characteristics of these included studies are shown in Table 1.

**Table 1:** Characteristics of the included studies.

References	Study design	Sample size	Mean age (range)		Intervention		Treatment course	Outcome
		T/C	T	C	T	C		
Kuang et al., 2020 (21)	RCT	39/38	69.23 ± 8.34	69.35 ± 8.29	Escitalopram (10 mg qd) + Carvedilol + RT	Carvedilol (5 mg qd, 3 days; 10 mg qd, 87 days) + RT	3 months	①②③⑧
Jia et al., 2012 (22)	RCT	49/49	71.3 ± 5.45	70.6 ± 5.02	Escitalopram (5 mg qd, 3 days; 10 mg qd, 53 days; less than 20 mg qd) + RT	Sertraline (25 mg qd, 3 days; 50 mg qd 53 days; less than 200 mg) + RT	8 weeks	①④⑧
Jia et al., 2017 (23)	RCT	49/48	71.2 ± 5.8		Escitalopram (5 mg qd, 3 days; 10 mg qd, 39 days; less than 20 mg qd)	Placebo + RT	6 weeks	①②③⑤
Ding et al., 2017 (24)	RCT	60/60	70.34 ± 4.86	71.12 ± 5.09	Citalopram(10–20 mg qd) + RT	RT	6 months	①②④
Wang et al., 2021 (25)	RCT	96/95	78.14 ± 10.94	79.46 ± 9.98	Citalopram (10 mg qd) + RT	RT	6 months	⑦⑧
Fraguas, 2009 (26)	RCT	19/18	73.5 ± 5.4		Citalopram (20 mg qd, 3 weeks; 40 mg qd, 6 weeks) + RT	Placebo + RT	8 weeks	⑥
Angermann, 2016 (27)	RCT	185/187	62		Escitalopram (5 mg qd, 3–6 weeks; 10–20 mg qd 6–9 weeks) + RT	Placebo + RT	12 weeks	①②⑥⑧
Cao et al., 2020 (28)	RCT	75/75	76.1 ± 4.8	75.1 ± 4.5	Escitalopram (5 mg qd, 3 days; 10 mg qd, 39 days; less than 20 mg qd)	Placebo + RT	8 weeks	①②③⑤⑧

RCT, randomized controlled trial; RT, conventional treatment. ① LVEF; ② LVEFDD; ③ NT-proBNP; ④ HAMD-17; ⑤ HAMD-23; ⑥ MADRS;⑦ GDS; ⑧ adverse reactions.

### 3.2. Included studies quality evaluation

**Figure 2:**
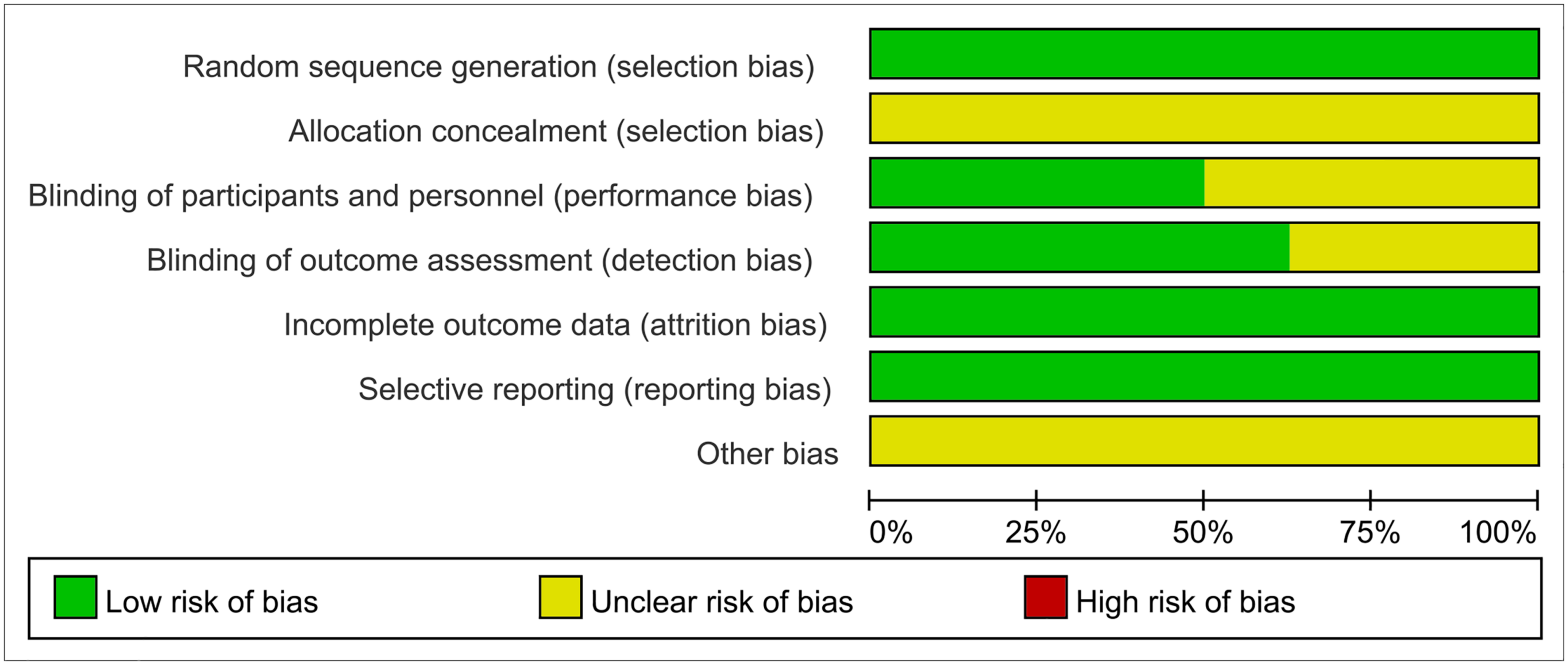
Risk assessment of bias.

**Figure 3:**
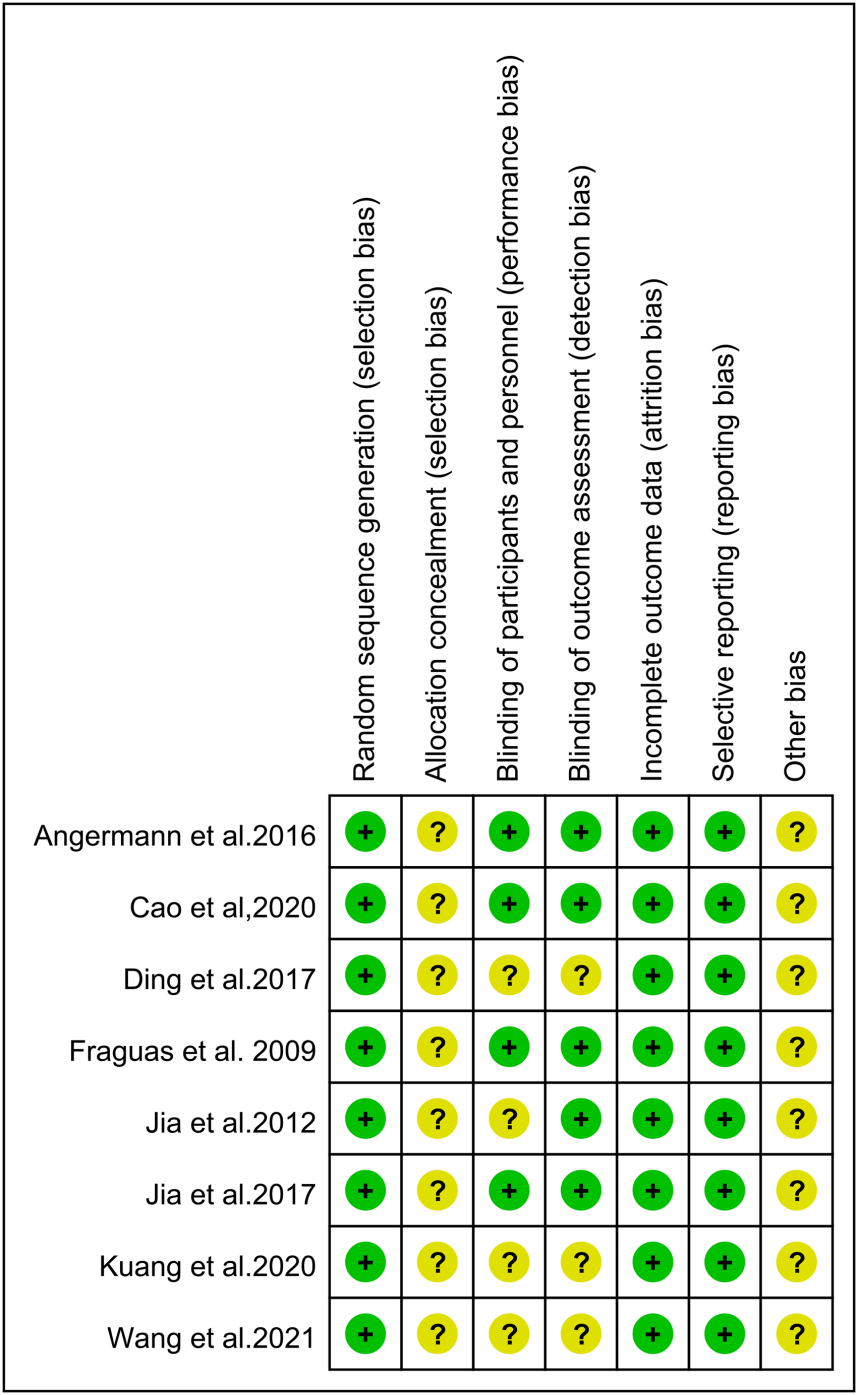
Risk of bias graph of included trials.

The inclusion of eight randomized trials were evaluated by the Cochrane risk bias assessment tool. All studies used the random method, in which three of the studies were conducted using the random number table method. All studies failed to mention the allocation concealment method. Five studies described the blind method, and three of the studies reported the double-blind method for patients. The quality and bias risks of studies are assessed in Figures 2, 3.

### 3.3. Meta-analysis results

#### 3.3.1. LVEF

**Figure 4:**
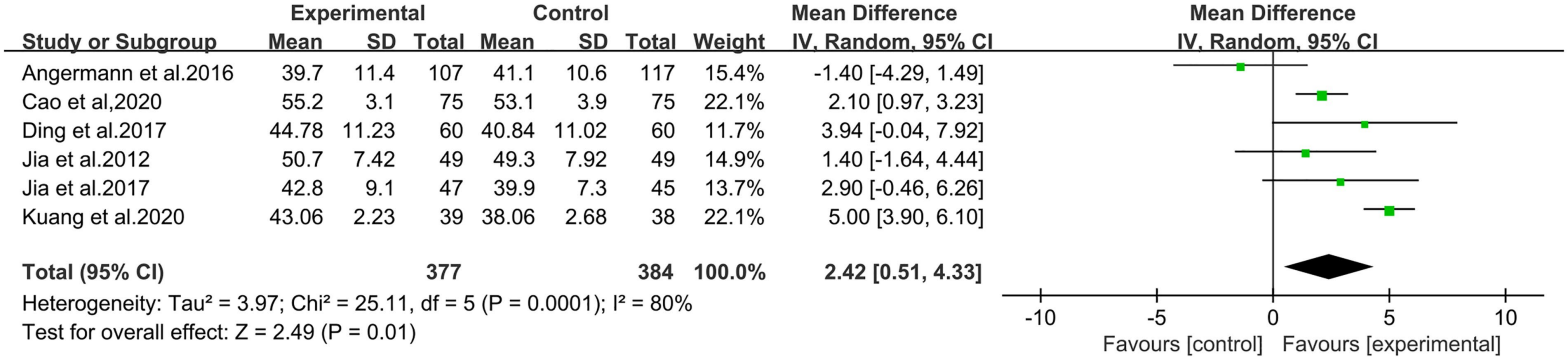
Meta-analysis for comparison of LVEF between the experimental and control groups.

There were six studies to report the LVEF results, involving 761 patients. The heterogeneity test showed the statistical significance (*p =* 0.0001, *I*^2^ = 80%), so we used the random effect model. We conducted a sensitivity analysis and removed the two studies. It was found that the heterogeneity was *I*^2^ = 47%, which might be related to its quality. The results of the meta-analysis showed that the difference between two groups was statistically significant (MD = 2.42, 95% CI: 0.51 to 4.33; *p* = 0.01; shown in Figure 4). This result indicated that the experimental group was better than the control group.

#### 3.3.2. LVEDD

**Figure 5:**
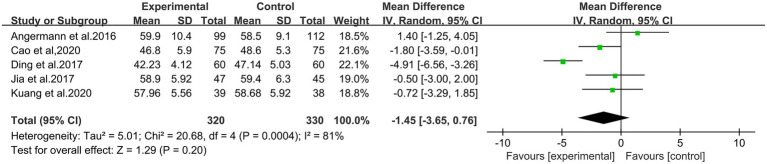
Meta-analysis for comparison of LVEDD between the experimental and control groups.

There were five studies to report the LVEDD results, involving 650 patients. The heterogeneity test showed statistical significance (*p* = 0.0004, *I*^2^ = 81%), so we used the random effect model. The results of the meta-analysis showed that the difference between two groups was statistically significant (MD = −1.45, 95% CI: −3.65 to 0.76; *p* = 0.20; shown in Figure 5). This result indicated that there was no significant difference between these two groups.

#### 3.3.3. NT-proBNP

**Figure 6:**
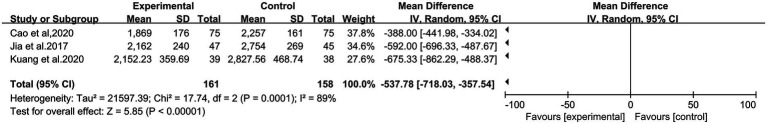
Meta-analysis for comparison of NT-proBNP between the experimental and control groups.

There were three studies to report the NT-proBNP results, involving 319 patients. The heterogeneity test showed statistical significance (*p* = 0.0001, *I*^2^ = 89%), so we used the random effect model. The results of the meta-analysis showed that the difference between two groups was statistically significant (MD = −537.78, 95% CI: −718.03 to −357.54; *p* < 0.00001; shown in Figure 6). This result indicated that the experimental group was better than the control group.

#### 3.3.4. HAMD-17

**Figure 7:**
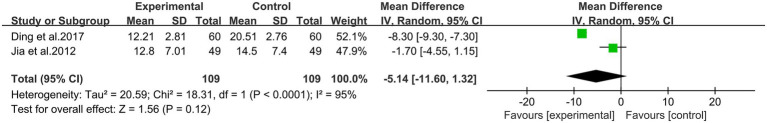
Meta-analysis for comparison of HAMD-17 between the experimental and control groups.

There were two studies to report the HAMD-17 score results, involving 218 patients. The heterogeneity test showed statistical significance (*p* < 0.00001, *I*^2^ = 95%), so we used the random effect model. The results of the meta-analysis showed that the difference between two groups was statistically significant (MD = −5.14, 95% CI: −11.60 to 1.32; *p* = 0.12; shown in Figure 7). This result indicated that the two groups had no significant difference.

#### 3.3.5. HAMD-24

**Figure 8:**
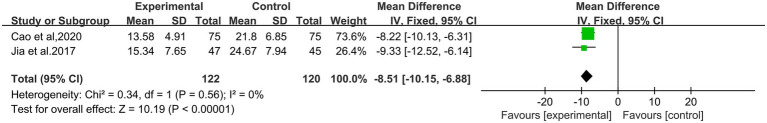
Meta-analysis for comparison of HAMD-24 between the experimental and control group.

There were two studies to report HAMD-24 score results, involving 142 patients. The heterogeneity test showed statistical significance (*p* = 0.56, *I*^2^ = 0%), so we used the random effect model. The results of the meta-analysis showed that the difference between two groups was statistically significant (MD = −8.51, 95% CI: −10.15 to −6.88; *p* < 0.00001; shown in Figure 8). This result indicated that the experimental group was better than the control group.

#### 3.3.6. MADRS

**Figure 9:**
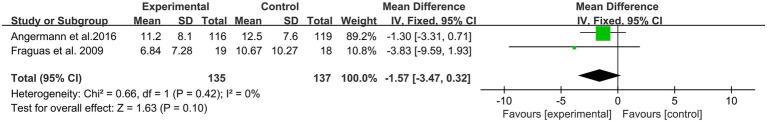
Meta-analysis for comparison of MADRS between the experimental and control groups.

There were two studies to report MADRS score results, involving 272 patients. The heterogeneity test showed the statistical significance (*p* = 0.042, *I*^2^ = 0%), so we used the fix effect model. The results of the meta-analysis showed that the difference between two groups was statistically significant (MD = −1.57, 95% CI: −3.47 to 0.32; *p* = 0.10; shown in Figure 9). This result suggested that the two groups had no significant difference.

#### 3.3.7. GDS

A study (25) using the GDS showed that both the observation and control groups had lower GDS scores after treatment than before treatment (12.53 ± 3.61 vs. 17.45 ± 4.31; 11.73 ± 2.96 vs. 17.22 ± 3.87, *p* = 0.000). The GDS score of observation group was significantly lower than that in the control group for moderate–severe depression patients (12.78 ± 2.46 vs. 14.96 ± 3.41, *p* = 0.012).

### 3.4. Publication bias

Since less than 10 articles were included, the evaluation of publication bias was not performed.

### 3.5. Safety

**Figure 10:**
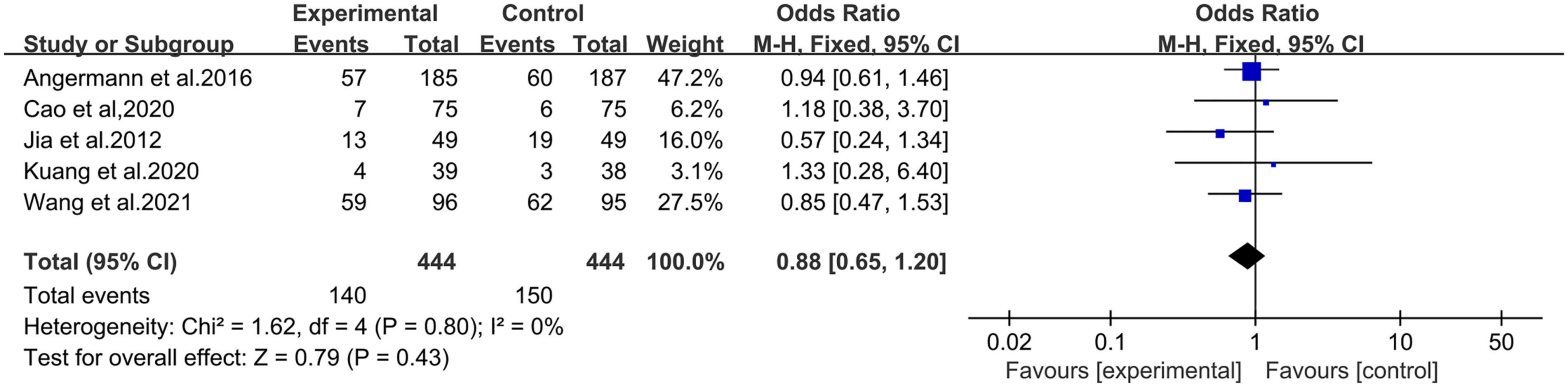
Forest plot comparing the levels of adverse reactions between the two groups.

Five studies were conducted to evaluate the safety and adverse drug reactions, such as nausea, vomiting, dizziness, fatigue, and insomnia occurred during treatment. There were 45 adverse reactions in the treatment group and 20 adverse reactions in the control group. The heterogeneity test showed the statistical significance (*p* = 0.80, *I*^2^ = 0), so we used the fixed effect model. The results of meta-analysis showed that there was no statistical differences between the groups (OR = 0.88, 95% CI: 0.65 to 1.20; *p =* 0.43; shown in Figure 10). Therefore, the experimental group did not increase the incidence of adverse reactions.

### 3.6. Grading of evidence quality

According to the further evaluation, the quality of evidence and the GRADE evidence profile was formed (in Table 2).

**Table 2:** The GRADE evidence profile for citalopram in the treatment of chronic heart failure combined with depression.

Certainty assessment						No. of participants (studies)	Effect	Quality of the break evidence break (GRADE)	
Outcomes	Risk of bias	Inconsistency	Indirectness	Imprecision	Other considerations		Absolute (95% CI)	Relative (95% CI)	
LVEF	Serious^a^	Not serious	Not serious	Serious^c^	None	761 (6 studies)	MD 2.42 higher (0.51 higher to 4.33 higher)	/	⊕ ⊕ ㅇㅇ low
LVEDD	Serious^a^	Not serious	Not serious	Serious^c^	None	650 (6 studies)	MD 1.45 lower (3.65 lower to 0.76 lower)	/	⊕ ⊕ ㅇㅇ low
NT-proBNP	Serious^a^	Not serious	Not serious	Serious^c^	None	319 (3 studies)	MD 537.78 lower (718.03 lower to 357.54 lower)	/	⊕ ⊕ ㅇㅇ low
HAMD-17	Serious^a^	serious^b^	Not serious	Serious^c^	None	218 (2 studies)	MD 5.14 lower (11.60 lower to 1.32 higher)	/	⊕ㅇㅇㅇ low
HAMD-24	Serious^a^	Not serious	Not serious	Serious^c^	None	142 (2 studies)	MD 8.51 lower (10.15 lower to 6.88 lower)	/	⊕ ⊕ ㅇㅇ low
MADRS	Serious^a^	Not serious	Not serious	Serious^c^	None	272 (2 studies)	MD 1.57 lower (3.47 lower to 0.32 lower)	/	⊕ ⊕ ㅇㅇ low

CI, confidence interval; MD, mean difference. GRADE Working Group grades of evidence. High quality: Further research is very unlikely to change our confidence in the estimate of effect. Moderate quality: Further research is likely to have an important impact on our confidence in the estimate of effect and may change the estimate. Low quality: Further research is very likely to have an important impact on our confidence in the estimate of effect and is likely to change the estimate. Very low quality: We are very uncertain about the estimate.

a All studies did not mention the allocation concealment method. Three study reported the double-blind method for patients. One study reported the single-blind method for patients.

b Wide variance of point estimates.

c Small sample sizes and not meet the optimal information size.

## 4. Discussion

In this study, we included a total of eight randomized controlled trials of the citalopram for elderly chronic HF combined with depression in a meta-analysis, involving 1,141 patients. This study preliminarily indicated that the citalopram would show a benefit in LVEF (MD: 2.42, 95% CI: 0.51 to 4.33, *p* = 0.01) and NT-proBNP (MD: −537.78, 95% CI: −718.03 to −357.54, *p* < 0.0001) associated with people of elderly chronic HF combined with depression. However, there was no positive effect on improving LVEDD (MD: −1.45, 95% CI: −3.65 to 0.76, *p* = 0.20). It was found that the citalopram could improve the depression instruments HAMD-24 (MD: −8.51, 95% CI: −10.15 to −6.88; *p* < 0.0001) in patients with elderly chronic HF and depression, while did not exhibit a statistically significant improvement in HAMD-17 (MD: −5.14, 95% CI: −11.60 to 1.32; *p* = 0.12) and MARDS (MD: −1.57, 95% CI: −3.47 to 0.32; *p* = 0.10).

The heterogeneity of HAMD-24 and MARDS was small (*I*^2^ = 0%), which was no significant correlation with drugs or oral methods. The heterogeneity of the HAMD-17 was large (*I*^2^ = 95%). Unfortunately, the cause of heterogeneity had not been found. Moreover, the association needs to be included more studies to explore. The study of GDS score was significantly lower in moderate–severe depression patients of the citalopram treatment group than in that of control group after treatment (25). The results of the meta-analysis proved that the citalopram treatment for depressed patients with chronic HF had a benefit in LVEF, NT-proBNP, and HAMD-24, while data indicated there was no good effect on LVEDD, HAMD-17 and MADRS.

In addition, in terms of safety evaluation, four studies reported that patients had no adverse reactions during the medication. Seven studies reported that patients experienced nausea, vomiting, dizziness, fatigue, and insomnia during medication, which may be related to the patients’ physical condition or underlying disease. However, the difference between the two groups was not statistically significant. Therefore, it was shown that the citalopram had a better safety profile in the treatment of CHF combined with depression.

The primary outcomes of the meta-analysis were changed in cardiac function and whether depression improved. As we know, LVEF, LVEDD, and NT-proBNP have proven to be important indicators for assessing cardiac function. Due to the different criteria for evaluating depression in the included articles, including HAMD-17, HAMD-24, MARDS, and GDS, the improvement of depression was analyzed separately. However, this adversely affected the analysis of the reliability of the results through Meta-analysis. Future studies of more uniform depression criteria will provide the potential for analysis of these outcome indicators. Citalopram has the effects of inhibiting platelet function, promoting endothelial stability and anti-inflammation (29, 30). It can improve cardiac function by relieving depression in patients with HF. This beneficial effect is a reduction in myocardial oxygen consumption (31), resulting in a significant improvement in LVEF and a significant reduction in NT-proBNP levels.

This study provides preliminary evidence that the citalopram can alleviate depression and improve cardiac function in patients with HF. In the included studies, the patients’ depression may be relieved through standardized medical treatment of HF. As a result of taking antidepressants at the same time, the patient’s depression status has been improved to a greater extent. Reduction of depression, improved mood and quality of life, promote the recovery of cardiac function. The relationship between chronic HF and depression is mutual.

The common pathophysiological mechanism is related to the following: endothelial function (32, 33); inflammation, including interleukin (IL)-6, C-reactive protein (CRP) and tumor necrosis factor alpha (34–36). Unfortunately, a number of articles included in this study about the inflammatory factor test were not sufficient for a meta-analysis. So, the study did not provide relevant data to observe the changes in inflammatory factors.

At present, some strategies may be beneficial for depressed patients with chronic HF, such as exercise programs, cognitive behavioral therapy and antidepressant medication (37). Exercise therapy significantly improves the symptoms of depression in patients with chronic HF, and improves both mental and physical health (38). Meanwhile, the cognitive behavioral therapy (39) has been shown to improve mental health outcomes in patients with HF. Due to the intersection of cardiac and psychological symptoms, it is not clear whether antidepressants can improve cardiac function. There are no clear findings on the choice of medication for patients with depression in HF. Currently, the citalopram is safe (30, 39) for treating patients in HF with depression, and no obvious adverse reactions have been found, which is consistent with our meta-analysis.

However, the inclusion of two studies (26, 27) showed that the citalopram did not have more effective over placebo in the treatment of depression. This inconsistency with the results of the meta-analysis may be related to several reasons. Firstly, populations domestically and internationally may be closely related to the results of the experiment, which had differences in drug sensitivity and tolerance. Secondly, the level of depression in the patient may be influenced by other underlying factors during treatment. For example, interpersonal communication, exercise, etc. To reduce the impact of potential factors on the results, we will try to explore the daily routine of patients of different races or ethnicities enrolled in future studies.

We have only studied the efficacy of the citalopram in the treatment of depression in the elderly with chronic HF, and will expand the scope to study young people in the future. In the past, we paid more attention to cardiac physiology and often ignored social and psychological factors (40, 41).

The relationship between the cardiac function and psychology studied vigorously. Regarding the improvement of depression in patients with HF by the citalopram, we can focus on the physical and chemical indicators of the brain in the future to obtain the meaningful answers from them. During the research process, we found that nursing staff (42) played an important role in alleviating patients’ depression. The goal in the future is not only to rely on drug treatment, but also to alleviate the suffering of patients from multifaceted interventions.

## 5. Limitations

However, this study has some limitations. Firstly, none of the included trials explained the method of allocation concealment, and most of them were published in Chinese, which adversely affected the reliability of the results. Secondly, fewer indicators were included in the English articles and we tried to contact the authors by email, but unfortunately without success. Thirdly, the chemical structure of the citalopram differs in the included studies, and this would have a biased effect on the reliability of the results.

## 6. Conclusion

According to this study, the citalopram treatment for depressed patients with HF have a benefit in LVEF and NT-proBNP, improve HAMD-24 and GDS, while data needed to verify the relative benefits of LVEDD, HAMD-17, and MADRS. More large-scale, multicenter, long-term, randomized, and double-blind clinical trials are needed to demonstrate this conclusion in the future.

## Data availability statement

The original contributions presented in the study are included in the article/Supplementary material, further inquiries can be directed to the corresponding authors.

## Author contributions

KC and JZ directed and supervised the study. LY conceived this study, analyzed the data and drafted the manuscript. YA, YX, and AG extracted the data. LY and QX rechecked the data. BW and HL provided valuable suggestions for article. All authors contributed to the article and approved the submitted version.

## Funding

This study was supported by the CACMS Innovation Fund (Grant No. CI2021A00915) and National Natural Science Foundation of China (Grant No. 81573817).

## Conflict of interest

The authors declare that the research was conducted in the absence of any commercial or financial relationships that could be construed as a potential conflict of interest.

## Publisher’s note

All claims expressed in this article are solely those of the authors and do not necessarily represent those of their affiliated organizations, or those of the publisher, the editors and the reviewers. Any product that may be evaluated in this article, or claim that may be made by its manufacturer, is not guaranteed or endorsed by the publisher.
