# Supplementary material for: Citalopram in the treatment of elderly chronic heart failure combined with depression: A systematic review and meta-analysis
Source: Front Cardiovasc Med. 2023 Feb 1;10:1107672. doi: 10.3389/fcvm.2023.1107672 (PMC9933506; doi:10.3389/fcvm.2023.1107672)
Supplement: Supplementary file 1 [file Table_1.DOCX]

Supplementary Material

Supplementary Table 1  Search strategy for the Embase database

| **Search Query** | |
| --- | --- |
| #10. | #3 OR #6 AND #9 |
| #9. | #7 OR #8 |
| #8. | depressive symptom*[Title/Abstract] |
| #7. | "Depression"[Mesh] |
| #6. | #5 OR #6 |
| #5. | ((cardiac failure[Title/Abstract]) OR (heart decompensation[Title/Abstract])) OR (myocardial failure[Title/Abstract]) |
| #4. | "Heart Failure"[Mesh] |
| #3. | #1 OR #2 |
| #2. | (cytalopram[Title/Abstract]) OR (escitalopram[Title/Abstract]) |
| #1. | “Citalopram”[Mesh] |
